# Process evaluation of the ‘Lafiyan Yara’ project on enhancing access to HIV testing services using existing community structures in Nigeria

**DOI:** 10.1186/s12889-024-18045-4

**Published:** 2024-02-27

**Authors:** Olujide Arije, Rachel Titus, Idowu Omisile, Aisha Dadi, Danjuma Garba, Omoregie Godpower, Jennifer Anyanti, Omokhudu Idogho, Emeka Okeke, Carmen Roebersen, Eliane Vrolings, Adedeji Onayade

**Affiliations:** 1 Institute of Public Health, Obafemi Awolowo University, Ile-Ife, Nigeria; 2 Society For Family Health, Abuja Nigeria Public Health, Abuja, Nigeria; 3 Taraba AIDS Control Agency, Jalingo Taraba State, Jalingo, Nigeria; 4 Aidsfonds, Amsterdam, The Netherlands

**Keywords:** Lafiyan Yara, Process evaluation, HIV testing services, Taraba State, Nigeria

## Abstract

**Background:**

The Lafiyan Yara Project aimed to increase demand for HIV counselling, testing, treatment, and prevention services among pregnant women and children in Taraba State, Nigeria. Implemented from 2019 to 2021, the project utilized existing community structures, including traditional birth attendants, village health workers, and patent and proprietary medicine vendors, for mobilization. This study assessed the project’s activities, contributors, relevance, effectiveness, and efficiency.

**Methods:**

The process evaluation was conducted using focus group discussions and key informant interviews with beneficiaries, community leaders, project staff, health facility personnel, and government officials. Data analysis employed framework analysis.

**Results:**

The Lafiyan Yara project was reported to have achieved notable successes, including increased HIV testing rates among children and pregnant women, improved linkage to care services, reduced mother-to-child transmission of HIV, increased HIV/AIDS awareness and knowledge, and enhanced community engagement and support. Challenges identified included insufficient funding for community mobilizers, training needs for health workers, and inadequate availability of test kits at health facilities. Confidentiality and stigma issues arose during community mobilizations. A key lesson learned was the importance of a comprehensive HIV care approach, emphasizing testing and ensuring support for individuals testing positive.

**Conclusions:**

The project’s approach of leveraging community structures to create demand for HIV services among women and children proved effective, provided proper linkage to care for those testing positive. Addressing stigma and involving husbands/fathers in the community approach are crucial for improving outcomes.

**Trial registration:**

IPHOAU/12/1384.

## Background

Nigeria has one of the highest number of persons living with HIV in sub-Saharan Africa with an estimated 1.9 million people living with HIV (PLWHIV) in 2020 [1]. This makes Nigeria the third-largest country in terms of HIV burden worldwide [2]. The prevalence of HIV among adults aged 15–49 years is 1.3%, and 0.1% among children aged 0–14 years, while the prevalence rate among adult females is 1.8%, compared to 1.0% among adult males [3]. Recent studies indicate that the estimated HIV prevalence among pregnant women in Nigeria stands at approximately 7.22% [4]. Furthermore, Odafe et al. [5] estimated that in 2017, approximately 140,000 children aged 0–14 years were living with HIV in Nigeria, but only 35% had been diagnosed and were receiving antiretroviral therapy. These statistics highlight Nigeria’s significant HIV prevalence in sub-Saharan Africa. There has been some progress in HIV management and prevention in Nigeria, with a 39% decline in new HIV infections since 2010, attributed partly to improved access to testing and treatment [1]. In 2021, approximately 1.7 million people living with HIV in Nigeria were receiving ART [1]. Despite this, significant efforts are still needed to combat the HIV epidemic. Expanding access to testing, treatment, and prevention services remains a crucial priority in addressing this public health crisis in Nigeria.

One effective method to control the spread of HIV is increasing awareness of personal HIV status through testing services [3]. However, HIV testing coverage among pregnant women in Nigeria is low, with only about 28.5% of them being tested, even in high-prevalence regions [6]. This lack of testing leads to limited awareness of HIV status, increasing the risk of transmission for adults and children, including mother-to-child transmission. Nigeria has implemented several programs to expand access to HIV testing, with a specific focus on pregnant women to prevent mother-to-child transmission (PMTCT) of HIV. One notable initiative is the integration of traditional birth attendants and primary health centers in the HIV testing process, known as the TAP-In model [7]. This approach aimed to increase HIV Counseling and Testing (HCT) uptake among pregnant women by leveraging the accessibility and community trust in traditional birth attendants, alongside the formal healthcare system's resources and expertise. The TAP-In model showed positive impacts on HCT uptake among pregnant women in intervention groups, indicating its potential effectiveness in enhancing access to HIV testing for pregnant women in community birth centers across Nigeria.

Challenges in HIV testing services stem from client preferences for privacy, anxiety about results, and fear of stigma [8–10]. To overcome these barriers, targeted outreach campaigns are crucial. Community-based health workers have proven effective in delivering targeted health interventions [11, 12] and play a key role in conducting these campaigns. For HIV, they can enhance campaign impact, create a supportive environment, and encourage individuals to seek testing. Research shows that involving community health workers improves testing rates and linkage to care [13, 14]. By leveraging their relationships and knowledge, community-based health workers promote testing benefits, provide information, organize events, and serve as trusted guides. Their involvement can bridge the gap between challenges and successful testing services, ensuring privacy, trust, and improved outcomes.

In Nigeria, many women still prefer to seek care from traditional birth attendants (TBA) rather than conventional health facilities. Reasons for this preference includes the perceived higher efficacy of traditional medicines [15] age-long cultural practices [16]easier to access TBAs compared to skilled birth attendants (SBAs) [17], lower cost of making TBAs a more affordable option for many women [18]. Utilization of TBA poses challenges for accessing HIV counselling and testing (HCT) services since they do not typically offer this service. When a child falls ill within the community, the initial point of contact is typically the patent and proprietary medicine vendors (PPMV), while volunteer village health workers, residing among community members, are also easily accessible. PPMVs are individuals registered with the relevant government authority to dispense over-the-counter drugs and medications in their local communities. These community structures offer potential human resources for mobilizing community members to utilize HIV testing services (HTS). Also, village health workers (VHW) live within the same neighbourhoods/communities as their potential clients, and can be easily accessed, at least, as the first point of contact for basic health care.

The Lafiyan Yara project was conceptualized as a context-specific, community participatory intervention. It proposed to guarantee the rapid identification and linkage of pregnant women and children less than 15 years of age living with HIV in selected Local Government Areas (LGAs) of Taraba State, Nigeria, to HTS and PMTCT services in state government-owned facilities. The theory of change of the Lafiyan Yara intervention was that to facilitate early detection of HIV, increased access to antenatal care (ANC) services by pregnant women and quality delivery services by health workers would enhance exposure to HIV counselling and testing and PMTCT services which consequently would eliminate new infections in babies. Similarly, improved linkages between informal and formal health structures in Taraba state would amplify findings of new HIV positive cases, increase antiretroviral uptake, increase the number of virally and ultimately suppressed women and children living positively and invariably reducing mortality among target groups. The uniqueness of this intervention lay in its emphasis on demand creation through community mobilizers, which was not a primary focus in previous projects in the area intervention area (personal communication).

This study was a process evaluation designed to explore the implementation of different types of community health workers as intervention models within the Lafiyan Yara project, investigating how these variations influence program outcomes. Its primary objective was to document evidence regarding the project’s implementation process, including the identification of successful components and areas needing improvement, while exploring the underlying reasons. The evaluation assessed the extent to which the intervention models were implemented as intended, their uptake, and the challenges encountered. Additionally, the study aimed to determine whether the interventions achieved their intended outcomes and identify any unintended consequences. It also sought to evaluate the level of community engagement, including the participation of children, and generate actionable recommendations for enhancing the program.

## Methods

### Study location and setting

Taraba state has an HIV prevalence of 2.9% which is the highest in the Northeast geopolitical zone and the second highest in the country [3]. Antenatal care attendance is 44.5%, which is lower than the average for the northeast geopolitical zone of 62.4% [19]. The estimated proportion of population that are pregnant women and children below 15 years old in the state are 5% and 41% respectively. The drivers of the HIV epidemic in Taraba state include norms that promote multiple concurrent sexual partnerships, low risk perceptions, low awareness of HIV, and poor literacy rates. There are also limited or no donor funded HIV interventions in Taraba (personal communication), leaving a gap in the continuum of care for HIV.

The Lafiyan Yara project was carried out in eight selected LGA across the three senatorial districts in the Taraba State, Nigeria, namely: Taraba North senatorial district (Jalingo, Zing & Karim Lamido LGA); Taraba South senatorial district (Wukari LGA); and Taraba Central senatorial districts (Gassol, Bali, Gashaka & Sardauna LGA). However, the multi-method intervention evaluation, of which this process evaluation was a part, was limited to four intervention LGAs. The other methods included a quasi-experimental study [20] and a cost effectiveness analysis.

### The intervention

Different models of community mobilization for HTS were used in the four study LGAs. Traditional birth attendants (TBAs) model was implemented in Bali, Village Health Workers (VHWs) model in Gashaka LGA, Patent and Proprietary Medicine Vendors (PPMVs) model in Zing LGA, and in Jalingo LGA a combination of all the three models was implemented. Lau LGA was the control LGA and had none of the interventions. For the purpose of this intervention, PPMVs are persons registered with appropriate government agency to sell off-the-counter drugs and medications within their communities, TBAs are women living within communities, who have no formal health training but provide midwifery services to pregnant women, while VHWs are individuals living within communities, usually having up to secondary level education but no formal health training, who volunteer, and are trained to provide specified community health services to members of their communities. The implementation component of Lafiyan Yara was carried out by the Society for Family Health, Abuja, Nigeria, while the research component was carried out by the Institute of Public Health, Obafemi Awolowo University, Ile-Ife, Nigeria.

The mobilizers had the task of locating pregnant women, informing them about HIV Testing Services (HTS) for themselves and children in their households, and guiding them to nearby public health facilities for an HIV test. Each health facility involved in the project designated a focal person to whom beneficiaries could be referred. Furthermore, community volunteers, typically employees of local organizations, were recruited to gather and summarize data on referrals and tests from the health centres each month, aiding in the project’s ongoing monitoring and evaluation. Incentives the community mobilizers were structured as follows: Traditional Birth Attendants (TBAs) and Village Health Workers (VHWs) initially received ₦12,000, which was later increased to ₦30,000 to align with the national minimum wage. Patent and Proprietary Medicine Vendors (PPMVs), who generally ran their own drug stores and did not make home visits, were excluded from this incentive. However, all community mobilizers received a ₦100 incentive for each successful referral, which occurred when the referred individual undergoes HTS at a health facility.

### Study design and study population

The process evaluation adopted an exploratory approach using qualitative data collection methods to facilitate an understanding of the planning process, document what activities were undertaken, and highlight the key factors that led to the program outcomes. Focus group discussions (FGDs) were held with currently pregnant and recently delivered women (within the last one year). We also held children’s workshop with children in the 10–15 years’ age group (Table 1). In the children’s workshops, a modified version of focus group discussions was employed, specifically adapted to align with the comprehension and interest levels of children. These sessions were conducted in an engaging and playful manner, ensuring that discussions about research topics were both fun and accessible for the children. This approach facilitated active participation and meaningful contributions from the children in a comfortable and age-appropriate setting. Participants in the FGD and children’s workshop in were identified and invited with the assistance of community mobilizers who were familiar with community members. Also, local community leaders who were more familiar with the community members were asked to assist in engaging eligible participants that had been so identified. Homogeneity of the groups was ensured in terms of age and sex as with each FGD session. We also held FGD sessions with community mobilizers who were involved in the implementation of the intervention. Key informant interviews were held with selected community leaders/gate keepers, Lafiyan Yara project staff, health facility focal persons workers, and State and LGA health officials. In all, 138 persons participated in 12 FGDs while 23 persons participated in the KIIs.

**Table 1:** Categories of study participants

Type of Participant	Data collection method	No. of Sessions
Children (Female)	Children’s workshop	4
Children (Male)	Children’s workshop	5
Community Mobilisers (Male & Female combined)	FGD	4
Women who had a baby less than a year last year	FGD	4
Women who were currently pregnant	FGD	4
Member of relevant community-based organization	KII	4
Health facilities focal persons	KII	4
Interview with Community leaders/gatekeepers	KII	4
Interviews with Taraba AIDS Control Agency staff (State level)	KII	2
Interviews with selected LGA officials	KII	4
Interviews with the project staff	KII	5
**Total**		**44**

### Study tools

Study tools were designed for each type of study participant namely: FGD Guide for currently pregnant women/recently delivered women; Children’s workshop guide for children 10–15 years; KII guide for community leaders and gate keeper; KII guide for Lafiyan Yara project staff; KII guide for health workers; and KII guide for selected State and LGA health officials. The study tools were translated from English to the local language (Hausa Language) and back translated into English to assure quality. Research assistants were trained using both the English and Hausa versions of the tool. The tools were pre-tested on the field among participants who were not included in the final evaluation.

### Data collection

Access to the community and recruitment of participants for the process evaluation was facilitated by the existing relationships created by the Lafiyan Yara project. Permission for data collection sought from the Taraba State Ministry of Health. Eight male and female research assistants were recruited for the data collection comprising of both males and females. The minimum criteria for selection for researchers were post graduate qualification in social sciences, public health or related field. Male research assistants conducted male only FGDs and ditto for female research assistants. They were provided a four-day training with the first three days spent familiarizing on the research methods and research tools using didactic methods and role-play, while the last day was for pretesting of the tools, and for feedback. The feedback from the pretesting was used to revise the tools. The training ensured familiarity with the instruments and techniques to be used and will also. All FGD session were conducted by pairs of moderators and notetakers, while KII were conducted by single interviewers. All sessions were recorded digitally after taking permission from the study participants. Quality control mechanisms instituted during data collection included daily debriefing before and after data collection, concurrent transcription by separate transcribers with vetting by the supervising team lead.

### Data analysis

The recorded sessions were transcribed verbatim and any interview/session conducted in Hausa, was transcribed directly into English Language. A model transcription protocol was developed and shared with all transcribers to ensure uniformity. However, proofreading of all transcripts was done by the interviewers to ensure their correctness and fidelity to the recordings. Analysis of the audio transcripts from the interviews and discussions was done using the ATLAS.ti 9 software for coding based on a Framework Analysis that prioritized matters on relevance, effectiveness, efficiency, impact, coherence, and sustainability of the project (Table 2). Coding was done by two expert coders who are certified ATLAS.ti users. The coding was used to breakdown the transcribed data into units of meaning or concepts which were subsequently categorized and labelled. Following more refinements, recurrent, dominant and divergent opinions were identified by linkages and integration of categories around a core of emerging constructs which was used to tell a full story of the selected intervention strategies.

**Table 2:** The process evaluation criteria framework

Evaluation Criteria	Main Research question
Relevance	Did the intervention do the right things in the key activities that were carried out.
Effectiveness	Did the intervention achieve its objectives?
Efficiency	How well were the resources used?
Impact	What difference did the intervention make?
Coherence	How well does this intervention fit in with other interventions in the study area, region or country?
Sustainability	Will the benefits of the intervention last?

## Result

The study results of the process evaluation are presented starting with the background characteristics of participants, key project activities, and the roles, contributions of key project contributors, and subsequently the evaluation’s thematic areas. These include the program’s effectiveness in enhancing the uptake of HIV testing services, the roles and contributions of various stakeholders, the impact of the project on HIV awareness and stigma, its efficiency in resource utilization, and finally, the program’s sustainability, exploring the potential for its ongoing success beyond the project’s life cycle.

### Background characteristics of participants

One hundred and thirty-eight persons participated in the focus group discussions. As shown in Table 3, majority were younger than 15 years old (43.5%), female (69.6%), single (50.7%), Christians (58.0%), had highest level of education as secondary school (58.7%), and students (55.1%). The participants included children, community mobilizers, currently pregnant women and women who recently delivered. Twenty-three people participated in the key informant interviews. The highest proportion were 45 years or older (43.5%), and majority were males (73.9%), married (73.9%), and had tertiary education (73.9%) [Table 4]. The KII participants included State and local health official, community leaders, community volunteers, health facility focal persons, and project staff.

### Key project activities

The Lafiyan Yara project was implemented in various communities in Taraba State, Nigeria, to promote HIV testing and linkage to HIV care for children and pregnant women. Key activities carried out in the project included advocacy visits to stakeholders at state, and local government and community levels by the Lafiyan Yara staff to introduce the intervention, community mobilization for HIV testing, health education, counselling, and referral for HIV testing, and regular awareness/outreach programs. Advocacy was also carried out to encourage men to allow their wives to visit facilities for HIV testing and antenatal care. Health education, counselling, and referral were provided by community mobilizers and health facility focal persons. Positive cases were linked to care, retained in care, and provided with social and economic support. According to LGA staff,

*“… … in the Lafiyan Yara project they have engaged certain volunteers like case managers … in the past I used to know some people when they come and get tested positive they will run away they will probably not given access medication but with the coming of the Lafiyan Yara project and the engagement of volunteers, a lot of these types of positive clients are being tracked back to the facility and they are also being given adherence counseling you know with the medication counselling and what have you so this has really been one of the successes that we see that Lafiyan Yara project… in our communities.” – Local AIDS Control Agency (LACA) Staff*.

**Table 3:** Background characteristics of FGD participants

Characteristics	Freq. (*N* = 138)	
**Age group**		
< 15 years	60	43.5
16–25 years	25	18.1
25–34 years	22	15.9
35–44 year	19	13.8
> 45 years	10	7.2
Missing	2	1.5
**Sex**		
Female	96	69.6
Male	42	30.4
**Marital status**		
Married	66	47.8
Single	70	50.7
Widow/Divorced	2	1.5
**Religion**		
Christian	80	58.0
Muslim	58	42.0
**Educational level**		
Non/arabic	10	7.2
Primary	23	16.7
Secondary	81	58.7
Tertiary	24	17.4
**Occupation**		
Farming	10	7.2
Civil servant	3	2.2
Trader/Self employed	36	26.1
Students	76	55.1
Comm health workers	5	3.6
Others	6	4.3
**Missing**	2	1.5
LGA		
Bali	36	26.1
Gashaka	30	21.7
Jalingo	37	26.8
Zing	35	25.4
**Participant type**		
Children	62	45.0
Community mobilizers	21	15.2
Currently pregnant women	30	21.7
Women who recently delivered	25	18.1

**Table 4:** Background characteristics of KII participants

Characteristics	Freq. (*N* = 23)	
**Age group**		
25–34 years	2	8.7
35–44 year	4	17.4
> 45 years	10	43.5
Missing	7	30.4
**Sex**		
Female	2	8.7
Male	17	73.9
Missing	4	17.4
**Marital status**		
Married	17	73.9
Single	2	8.7
Missing	4	17.4
**Religion**		
Christian	7	30.4
Muslim	11	47.8
Missing	5	21.8
**Educational level**		
Tertiary	17	73.9
Missing	6	26.1
**Occupation**		
Civil servant	11	47.8
Project staff	4	17.4
Others	3	13.0
Missing	5	21.8
**LGA**		
Bali	4	17.4
Gashaka	4	17.4
Jalingo	4	17.4
Zing	4	17.4
State level	7	30.4
**Participant type**		
State health official	2	8.7
Community leaders	4	17.4
Community volunteers	4	17.4
Facility focal persons	4	17.4
Project staff	5	21.7
Local AIDS Control Agency Officer	4	17.4

### Key contributors and their roles

The success reported of the Lafiyan Yara project is largely attributed to the efforts of various key contributors. These include community leaders and members of traditional councils, community mobilizers (traditional birth attendants, patent medicine vendors, and village health workers), community volunteers, health workers, government officials, religious leaders, and program staff. Community leaders and members of traditional councils played a significant role in the program’s success by facilitating community entry and program acceptance. Representative comments reflecting this are as follow:

“…*the community [was] very important because without them embracing this program, it wouldn’t have been successful especially those concerned wouldn’t have been able to give out their consent.”* – Community mobilizer from Bali LGA.

“…*they followed through the hospital then through the traditional council, and traditional council connected them with people that brought the workers and where there is a hitch*, *the traditional council will intervene to ensure everything worked which helped them understand that this disease is real.”* – Community leader, Gashaka LGA.

“*I involved the Imam of all the mosques in my community, and I told them what to do, that they should be enlightening people* [about HIV], *and they did the same thing*.” – Community mobilizer.

Community mobilizers such as traditional birth attendants (TBAs), patent medicine vendors (PPMVs), and village health workers (VHWs) played a crucial role in the program’s success by providing counselling and referral services, ensuring linkage to care and management, and conducting awareness and mobilization activities. The following statements from interviewees corroborated mobilizers’ activities

“…*the Lafiyan Yara project has engaged three models, we have traditional birth attendant, we have the village health workers and patent medicine vendors. So, some of the local governments have only one model while some have 3, Jalingo has 3, I think Zing have only one model.*” State-level MOH official,

“…*the mobilizers…play a vital role” in convincing people to come for testing.”* - Community mobilizer from Bali LGA.

Community volunteers who are members of staff of community-based organizations in the intervention communities contributed to the program’s success by collating and validating data. They also collate all the referred and tested data from all the facilities and summarized it in one monthly summary form. A community volunteer from Jalingo explained as follows:

“W*e verify those lists with the community mobilizers in the facilities and make sure that the figures those community mobilizers give us…come[s] together with the figures at the facilities*.”

Health workers were essential to the program’s success by providing HIV testing and treatment services, coordinating and mobilizing the community, and supervising and monitoring the program’s activities. According to a community mobilizer from Jalingo:

“…*the major contributors are the project team and then followed by the facilities staff who actually made the room conducive for counselling and testing to get quality results.”*

They also reached out to church and mosque leaders to educate their congregation and encourage them to patronize the program’s community engagement events. In addition, they facilitated community entry and mobilization.

Government officials from the Taraba AIDS Control Agency (TACA) and the Ministry of Health ensured an enabling environment for the program’s success. According to a program staff:

*“In Taraba State we had significant support, we have the national steering committee both at the national and the state level, we have the committee members which comprises of Taraba stakeholders*”.

Finally, it was noted that program staff played a crucial role in the success of the Lafiyan Yara program by conducting awareness and mobilization activities, coordinating program activities, facilitating community entry.

### Program relevance

The Lafiyan Yara project was relevant to the HIV situation in the intervention location for several reasons. Firstly, it addressed key issues such as awareness creation and education. Secondly, the project provided care and support for children exposed to HIV. Thirdly, it included the education of pregnant women on preventing mother-to-child transmission. Finally, the project facilitated HIV referral and linkage for care. One pregnant woman expressed her satisfaction with the project as follows:

“*The sickness that another has will not be transferred to a child plus we are given instructions on when we can breastfeed our children. If a child is breastfeeding more than he should, he is able to contract it. But since we met people who have been helping us, we find out that our children are healthy leaving only the parents infected.”*

Another community leader stated that,

…*the Lafiyan Yara project filled a gap in previous projects by focusing on women and children, and corrected mistakes made in previous programs*.

The project was relevant to referral for HTS, as it helped to create demand for HTS services, resulting in increased utilization of the service. One participant stated,

“*Lafiyan Yara had helped in the creation of demand for HTS such that people were now visiting the center for testing by referral, including self-referral*.”

Pregnant women who tested positive had access to free drugs for health management and prevention to prevent the spread from mother to children. Furthermore, patent medicine store owners and TBAs had the opportunity to refer their clients for testing, and health facility staff were more actively engaged in testing and treatment.

“*It’s relevant in the sense that as I said it improved the HTS uptake in the facilities, it improves more case findings because some of the facilities were actually just sitting down, yes but with the support they got from Lafiyan Yara in terms the data capturing tools, test kits, the constant supervision, the constant improvement and the edge push, they’ve actually helped. Because even those people that were actually relapse from their medications now started coming out to actually receive treatment.” –* Program staff.

The house-to-house awareness and referral strategy, community outreach programs, and the implementation research component were identified as the most useful aspects of the HIV prevention project. According to a state-level Ministry of Health official, the research aspect of the project was particularly innovative. He said:

“*I found the research aspect more useful because this is innovation that has not been done before in the program. Demand creation is not something new, but the research component of this project that is trying to look at this model is very new to me and of course to the agency.”*

Also, the use of existing community structures and partnerships with health facilities for HIV services was another useful and relevant aspect of the project. This innovative approach, which included a research component, was expected to enable evidence-based decision-making in the future.

One aspect of the project that posed challenges was the burden of going to health facilities for testing. This burden discouraged some patients from accessing HIV services, often due to fear of stigmatization. Additionally, transportation issues were a significant barrier, especially for some pregnant women who lacked the funds or means to reach health facilities. Another problem was the frequent unavailability of testing kits and equipment at these facilities, leading to patients often leaving without being tested. The following statements reflected this issue:

*“… (what is) not appealing to them is the issue of walking, leaving their house to go and run tests, and people sometimes seeing them. I know they have been saying we should always keep it secret, but as a community which we are, we have always found it difficult and most people will secretly come in the night.”* - A health facility focal person.

“*Yes, lack of mobility and money because after creating awareness, people walk on foot to distant areas without mobility and even some of us do not have the means to go to the hospital.”* -A pregnant woman from Gashaka LGA.

“*One thing that did not appeal to them on the onset was the fact that demand was created, and they were mobilized to the facilities, but on getting to the facilities, the facility will not have a test kit to test them. So, they will have to start going back, and some of them like I said, they will come from far to these facilities to access services.”* - A program staff.

It was recommended by program staff that patients’ locations should be mapped and ART be delivered at their doorsteps to ease their transportation burden. One of the program staff said,

*…another burden they face is having to go to the facility for a refill, so with this current differentiated service delivery model, we are working to see how case managers will work closely with the facility to see that drug refills are taken to people at the community, especially the community members who are feeble*.

Finally, the short notice of service commencement and assessment visits was identified as another least attractive aspect of the project, which made it difficult for some community volunteers to participate. A community volunteer stated,

*The other aspects I can say that was not appealing to them is the short notice of information for any visit. Because when you, okay now, like as it is now, you called me yesterday and told me you will be coming and the next thing I could see is that this morning you are here and then having me on board*.

### Program efficiency

During discussions with clients, service providers, and project staff, it became evident that the resources allocated for various operations in the program were appropriately used. These resources included test kits, documentation and logistics, and financial resource management. According to a community mobilizer,

“*A lot of forms were printed, a lot of registers, things, materials were issued and I was aware of the palliative that was given to some persons, caregivers, and some positive clients.”*

Interviewees expressed a positive opinion regarding the judicious use of program sources. For example, one program staff member noted that,

*“…the resource usage, I think if I’m going to assess it, I’ll give it 100% because it’s efficiently used. As I’ve said from the beginning, we used it based on what it’s planned for. We used it, what is not in our plan we don’t even think about it nor using the money for a different activity, and you know we’re developing workers. We like working in the community. We workers we like contributing our quota to our communities we ensure that all that is planned with the resources are used for that…”.*

Despite this, some participants suggested that resources could be better utilized, including engagement with community pharmacists, the use of identification cards instead of referral forms, empowering community mobilizers to provide HIV testing services at the community level, and the appropriate utilization of mid-term evaluation reports. A LACA commented,

*“Well, that (judicious utilization of resources) depends on the outcome of the mid-term review of the project. What is the reality on the ground is what is going to be a pointer as to how best it can be properly utilized*”?

The interviewees also mentioned other challenges they experienced during the program implementation, including insufficient funds, non-availability or late supplies of test kits, supplies, and drugs, which led to short shelf life of some products, waste of certain resources, and challenges with transportation. According to a facility focal person,

“*The challenge is that sometime when the women come to the health facility to do their test, sometime they will not get the test because there are no enough test kits and some drugs that we will give them*.”

Interviewees frequently cited money-related challenges for clients, including paying for transportation to facilities, which can cost between thirty naira (₦30) to four thousand naira (₦4,000); payment for cards and treatment, which can cost between three hundred naira (₦300) to four thousand naira (₦5,000).

“*Yes, we do spend money for test and before you will start collecting the drugs, you will open a folder and we do that with money. …I spent over 4,000 naira.”* A pregnant client.

To meet these needs, some clients reported waiting for days to gather the necessary funds for drugs, while others borrowed money or took loans. According to a child,

“*Some of our parents have to borrow money from neighbors before they can take us to the hospitals*.” – Under – 15-year-old child.

### Program effectiveness

The interviews revealed that the intervention was effective in improving HIV testing and counselling (HTS) uptake in the targeted communities. The intervention was delivered by trained personnel, including traditional birth attendants (TBAs), village health workers (VHWs), and patent and proprietary medicine vendors (PPMVs), who conducted sensitization programs in schools, religious settings, healthcare facilities, and other public places. The personnel educated community members about HIV and encouraged them to visit healthcare facilities for testing and treatment. A pregnant woman who participated in a focus group discussion said,

“*They have helped us through awareness because we were ignorant when we were at home, but now they have informed us, and we feel better*.”

Other pointers of effectiveness as stated by the stakeholders were programme’s relevance to the community’s prevailing challenge, easy accessibility of testing and treatment, positive attitude of facility staff, and obvious improvement in health after treatment. However, the program faced some challenges, including concerns about privacy, poor quality of care received, and accessibility of some healthcare facilities. Despite these challenges, the program received positive feedback from the community. One community member commented,

“*Honestly, we have achieved a lot. The organization has helped by giving children milk, drugs, and other supplies provided at the hospital. Now they call to ask if there is a new thing they can benefit from*.”

However, a state-level staff member described a challenge they encountered regarding the quality of care received. He said,

“*We still experience a few instances of poor care, whereby clients complain and, based on a little assessment, we discover that some of these caregivers have not been trained for a long period of time. But many trainings are ongoing, people are realizing or are having knowledge of new innovations, new testing strategies, communication skills, and all this have helped in improving these services. So far, we have dealt with some pockets of cases except few from the local governments, which we are still working on*.”

Several factors influenced the program’s success, including support from community members, involvement of community mobilizers, attitude of community mobilizers, community testing, employment and training of workers, clients following due drugs and prescriptions, frequent checkups on status, availability of funding, house-to-house intervention supports, and increased awareness. A program staff member emphasized the importance of funding by saying,

“*Without funding, all these things I’m talking about are not possible. The funding we receive from our donor is one of the factors that influence it.”*

### Program impact

The study participants reported increased access to HIV testing services, care and treatment, and better follow-up/tracking and referral programs. One staff member of the Local Action Community on AIDS stated,

*It has had an impact, now the referral system is moving smoothly because if we refer, people are getting to the facility, and they are getting the referral treatment*.

The project effectiveness in increasing access to free drugs, boosted the morale of health workers, and changed community members’ perception of HIV. Describing her experience, a pregnant woman commented that,

Honestly, it has solved a lot of problems unlike before, when someone is sick but knows not what is wrong, but now, we have been helped to get proper treatment, and are no longer subject to ridicule by others and that has changed our thinking about service provision. I used to worry about what to do with my life and with my children if I died because we thought there was no cure. But now we have been helped.

The Lafiyan Yara project increased the knowledge of participants about HIV and changed their attitude towards the disease and those who might have contracted it. A woman in the FGD session said:

*Honestly, it has brought a lot of improvement. We were living in darkness now we are enlightened. Now we understand better what is happening. We can relate with people, enter anywhere, and no one will know.*

Participants also reported a change in stigmatizing attitudes towards HIV-positive individuals. Below is a representative quote:

“*Now they don’t despise us, we even laugh with them. Before, one becomes a subject of scorn and shame for being positive, but now we are even friends and we appreciate them*.” A recently delivered mother.

Other evidences indicative of success in achieving the project goals included the availability of drugs at healthcare facilities, increased awareness and sensitization about HIV, better delivery for children of infected mothers, and increased confidence among women and children. Community mobilizers also reported that there was improvement in the ways to handle women in labor who are expecting a baby to prevent the baby from contracting the virus. Furthermore, the program had good acceptance among community members, and there were testimonies of higher ANC turnout and healthy livelihoods of infected persons. According to a Local Government Health official,

*There’s a success since the evidence of the success is that people are aware, people are taking their drugs, people are moving freely now, no any problem again*.

Community members had a positive perception of the project, expressing their appreciation and support for the program’s benefits, including care for HIV-positive pregnant women and children, health education on HIV prevention and treatment during community outreach, house-to-house sensitization, counselling, referral to get free testing and drugs, increased knowledge of people’s status, and distribution of palliative to HIV patients during COVID-19 pandemic lockdown. The positive impact of the program prompted a high level of commitment from community leaders to mobilize targeted community members for testing, with one community leader instructing the town crier to announce that all children below 14 years of age should come for testing. They alluded that the program has contributed to the reduction in HIV spread and gradual eradication of HIV stigmatization. A pregnant woman expressed her gratitude, stating,

“*We are happy that you came, that you know we are important and you take care of us,” while a community mobilizer said, “… due to the house-to-house sensitization, we now know [who has HIV].”*

A child who participated in one of the children’s workshops also expressed appreciation, saying, “*They take the drugs to stay healthy and the people giving free medication are doing the right thing because they help their wellbeing.”*

The process evaluation did not reveal any negative or adverse unintended results/impact generated through the Lafiyan Yara project but this does not indicate an absolute/actual absence of such.

### Program coherence

The concept of program coherence refers to how well a program compares to similar projects in the community that provide the same intervention. In this study, participants discussed various HIV interventions, including case referrals, the supply of test kits, counseling, and PMTCT. When compared to other similar projects, the Lafiyan Yara program was considered to have several strengths, including a better follow-up system, a more specific focus on HIV status discovery among women and children, good community interaction and referral systems, a more targeted intervention focusing on households, the involvement of Patent and Proprietary Medicine Vendors (PPMVs), proper program monitoring, and the supply of HTS equipment. One community mobilizer noted the following:

*“… we don’t have any experience that among the other project of using PPMV that is what I’m saying right from the beginning; no any project that is using PPMV in terms of referring for HIV test and counseling except Lafiyan Yara. So there is more impact.”*

This demonstrates the unique strengths of the Lafiyan Yara program compared to other projects in the area. However, a respondent at the state level noted that it was too early to compare the results of the Lafiyan Yara program with other projects, given that the project was not yet concluded. According to him:

“*This project started in 2019, so we need to wait…for you to say this is the contribution of a project, usually, you need to do a kind of evaluation or a survey, currently a survey is done at random. We had the NARHS 2019 survey, so these are the population-based surveys to assess progress, so whatever is going to be judged based on that survey, so let us see maybe if another project is done, we can say when ‘Lafiyan yara’ project was in the State, they have contributed because it is not only them alone, other partners are also working, alright?”*

### Program sustainability

The sustainability of the program was a key concern for all stakeholders, including community members, mobilizers, facility focal persons, and project staff. While they expressed interest in continuing the program, they acknowledged that achieving sustainability would require joint efforts with the community, collaboration with other partners/donors, integration into the LGA structure, and proper planning. As a community volunteer noted,

“*I think it may be sustained even if this Society for Family Health is not taking care of it again, it may [be sustained] …with these personalities [i.e., community leaders, religious leaders, LACA, and others].”*

To ensure the program’s continuity, interviewees identified several key features, including enlightening husbands and parents on the importance of knowing their children’s HIV status and not stopping them from visiting health facilities, adequate provision and management of necessary resources, and proper training of all necessary personnel. A child participant in the children’s workshop emphasized the importance of educating parents for sustainability,

“*They should enlighten the parents to desist from stopping their children to go to the hospital as it will help them access their drugs.”*

## Discussion

The Lafiyan Yara project aimed to increase HIV testing services uptake at health facilities by creating demand through existing community structures/mechanisms such as PPMV, TBA, and VHW. This approach addressed a crucial gap in community-based HIV programming, specifically, the lack of demand creation for women and children using existing community structures. This focus on demand creation through community mobilizers was not a priority in previous projects, making this intervention unique. The project’s use of existing community structures, such as TBAs, and collaboration with health facilities to provide HIV services was a new approach to demand creation for testing and treatment. Additionally, the integration of an implementation research component in the intervention was considered innovative as it would provide evidence for the best model and enable scaling up the approach.

The research participants in this study reported the success of the Lafiyan Yara project in achieving its objectives, which they attributed to the involvement of various key stakeholders. The success of the program was made possible by the collaboration of community mobilizers, health workers, government officials, religious leaders, program staff, community leaders, and traditional councils. The multi-stakeholder approach proved effective in implementing health programs as it enabled the pooling of resources and expertise from different stakeholders. In the implementation of the program, community mobilizers played a critical role in providing counselling, referral services, awareness, and linkage to care. Health workers were instrumental in providing HIV testing and treatment services and overseeing program activities. Government officials, religious leaders, and program staff created an enabling environment that facilitated the program’s success. The involvement of community leaders and traditional councils facilitated community entry and program acceptance. The importance of community involvement in health interventions cannot be overstated. Research has identified community involvement as a crucial factor in increasing the success rates of health interventions [21]. Religious leaders have been found to play a significant role in facilitating community entry, mobilization, and overcoming cultural barriers in health programs. Additionally, community leaders and traditional councils are known to play key roles in facilitating community entry and acceptance of health programs, thus contributing to program success.

The Lafiyan Yara project aimed to reduce HIV spread through strategies such as HIV testing, counselling, and antiretroviral therapy, which have been proven effective in reducing HIV transmission [22]. The project successfully created demand for HIV testing services and improved the utilization of HIV Testing Services (HTS) in facilities. Additionally, the Lafiyan Yara project resulted in more case findings, improved engagement of health staff, and better data recording, similar to the results reported in other HIV prevention programs [23, 24]. Also, the success in creating demand for HIV testing and treatment services led to more people knowing their HIV status.

Community members appreciated the program and its benefits, such as care and health education, which are important factors for the success of public health interventions [25]. The most useful aspects of the project included house-to-house awareness, community outreach, and the implementation research component. These have been found effective in increasing access to and utilization of health services in other programs [26], while implementation research is useful for identifying barriers and facilitating program improvements [27]. The least attractive aspects, such as transportation challenges, lack of testing kits, and short notice of service commencement, are common challenges in resource-limited settings that can affect the effectiveness of public health interventions [28, 29].

Also, the research component of the Lafiyan Yara project was considered valuable and pertinent by government and state-level health officials. This was due to the potential for the research findings to serve as a foundation for evidence-based policy decisions regarding HIV intervention within the state. The inclusion of an implementation research component in the project provided an opportunity to assess the feasibility, effectiveness, and impact of the intervention. By evaluating the program’s outcomes and generating evidence on best practices, the research component will enable policymakers to make informed decisions and allocate resources to interventions with proven effectiveness. As such, the research component of the Lafiyan Yara project holds significant promise for enhancing the response to HIV in the state and contributing to the broader field of HIV intervention research.

Based on the study participants’ feedback, the resources allocated to the program were utilized efficiently, as evidenced by positive feedback from program beneficiaries. Efficient resource usage is critical for successful program implementation and better health outcomes, as demonstrated in various public health interventions [30, 31]. However, participants reported challenges faced during the program implementation, including insufficient funds, which is a common issue in public health interventions, particularly in resource-limited settings. Insufficient funds led to the late or non-availability of supplies and test kits, and transportation difficulties. Delays or non-availability of supplies could impact program efficiency and effectiveness, leading to missed opportunities for testing and treatment, as seen in other health interventions, particularly in HIV prevention programs [28, 29].

Financial constraints faced by clients were identified as significant barriers to accessing care. Such constraints have been consistently identified as a major barrier to accessing health services in various settings [32]. Costs of transportation, HIV tests, and treatment could prevent clients from accessing necessary care, worsening health outcomes as seen in other studies [32, 33]. Community mobilizers giving money to their referrals highlights the need for creative solutions to address financial barriers faced by clients. Addressing financial barriers through innovative strategies, such as subsidies or conditional cash transfers, has been shown to improve access to healthcare [34].

The effectiveness of the Lafiyan Yara project can be attributed to several factors such as community support and involvement leading to increased awareness and encouraging testing and treatment. Community involvement has consistently been linked to increased awareness and uptake of testing and treatment services in various health programs [35]. The use of Community mobilizers was crucial in engaging with community members and promoting the benefits of HIV testing and treatment, as seen in other settings [36]. Ensuring quality care for those who tested positive for HIV is an essential component of HIV prevention and treatment programs was also an important factor for the program success. However, challenges to program effectiveness which included privacy, accessibility, and quality of care need to be addressed for long-term effectiveness. To improve accessibility, it is essential to increase the number of healthcare facilities and community mobilizers. Enhancing the quality of care can be achieved by providing regular training and support to healthcare workers.

Generally, the feedback from the process evaluation was that the project had a positive impact on HIV prevention and treatment by improving social acceptance for those living with the disease and in addressing barriers to testing and treatment. The key finding of the program’s impact was that it has led to increased awareness of HIV testing, improved access to care and treatment, better follow-up, and reduced HIV prevalence in children. Corroborating evidence from the literature suggests that public health interventions that increase awareness and access to HIV testing and treatment improve outcomes, thus leading to reduced HIV prevalence in children [37]. Additionally, the project contributed to a change in stigmatizing attitudes towards HIV, reducing discrimination and social exclusion. Community-based interventions have been shown to reduce stigmatizing attitudes and improve social acceptance of people living with HIV [38]. The program increased knowledge and understanding of HIV and improved social acceptance for those living with the disease.

A major finding of the Lafiyan Yara project is that well-coordinated and aligned programs are more likely to be effective in achieving their goals. This is supported by previous studies that have demonstrated the effectiveness of coordination and alignment in public health programs [39, 40]. The Lafiyan Yara program exhibited unique strengths that contributed to its success, including enhanced follow-up, a specific focus on HIV status discovery, good community interaction, targeted intervention, involvement of community structures, proper monitoring, and supply of HTS equipment. While these specific strengths may not be directly corroborated or diverged by other studies, it is crucial to identify and leverage unique strengths of public health programs to improve their effectiveness and impact [41]. Further evaluation and surveys are necessary to fully assess the program’s effectiveness.

Community involvement and collaboration were found to be crucial for the sustainability of public health programs in the Lafiyan Yara project. This finding is supported by existing literature that recognizes the importance of community engagement in the success of public health interventions. Community involvement fosters ownership, enhances program success, and improves the likelihood of program sustainability [42, 43]. This study also highlights the necessary features for program continuity, such as adequate provision and management of resources, proper training of personnel, and parental education (for interventions that involve children). These factors have been identified as crucial for the success and continuity of public health programs in other studies intervention [44, 45]. Addressing critical areas during program implementation has also been found to improve long-term sustainability and positively impact community health [46, 47].

On the part of the community mobilisers, some felt the monitoring and evaluation forms they were required to complete were too cumbersome and needed to be reduced or made more manageable. To improve the monitoring and evaluation process for community mobilizers in future interventions, it is recommended to streamline and simplify the forms they need to complete, reducing the burden and ensuring ease of management. Additionally, the use of incentives should be carefully considered and implemented in a way that maintains the integrity of the project. It is important to establish regular training and support programs for healthcare workers involved in the project, enhancing their skills and knowledge to deliver high-quality care. Lastly, continuous evaluation and surveys should be conducted to assess and improve the program’s effectiveness, ensuring ongoing monitoring and refinement of the interventions. By implementing these recommendations, the project can achieve more efficient and effective outcomes while maintaining the integrity of the program and enhancing the quality of care provided.

### Lessons learned

Lessons learned from this intervention highlight the significance of involving community stakeholders from the planning stage to foster a sense of ownership within the community. The implementation of incentives effectively increased service uptake, with beneficiaries motivated by tangible rewards such as mosquito nets, soap, and monetary incentives. Providing adequate support to mobilizers, including increased stipends and access to transportation, proved crucial for successful implementation. A key lesson learned was the necessity of a comprehensive approach to HIV care, emphasizing the importance of not only increasing HIV testing but also ensuring that individuals who test positive receive the necessary care and support. This is particularly critical in areas with limited access to treatment, as underscored by one participant who stressed the need for ongoing support beyond testing. The findings of this study suggest that while the program was implemented efficiently, there are several challenges that need to be addressed to ensure effective program delivery and better health outcomes for clients. Addressing these challenges will require a concerted effort from all stakeholders, including program staff, clients, and policymakers. This may involve developing innovative strategies to address financial constraints faced by clients, improving the availability and distribution of essential supplies, and increasing funding for program implementation. By addressing these challenges, the program can be better positioned to achieve its goals of reducing HIV transmission and improving health outcomes for clients.

### Limitations

It is crucial to consider the limitations that might have influenced the findings in this study, such as selection bias from non-random participant selection, self-reporting bias in participant feedback, the cross-sectional design, and limited generalizability due to the unique context and setting. Also, this study adopted a purely qualitative approach, which, while insightful, could be complemented by quantitative methods for certain aspects of the analytical framework we used (e.g. effectiveness and impact). Quantitative findings from the Lafiyan Yara project have been detailed in a separate report [20]. Despite these limitations, the study offers valuable insights into leveraging existing community structures for HIV testing and counselling, emphasizing the importance of involving stakeholders, providing incentives, supporting mobilizers, and ensuring a comprehensive approach to HIV care. These lessons can inform future programming and contribute to the success of community-based HIV prevention interventions.

## Conclusion

The Lafiyan Yara project demonstrated the effectiveness of leveraging existing community structures and engaging multiple stakeholders to increase the demand for HIV testing services and improve access to care. The project’s innovative approach, which focused on demand creation through community mobilizers and collaboration with health facilities, has shown promising results in reducing HIV transmission and improving the lives of those affected by HIV. The project’s success can be attributed to community involvement, multi-stakeholder collaboration, and the integration of an implementation research component, which provided valuable insights for scaling up the approach. The findings from the Lafiyan Yara project highlight the importance of addressing barriers to testing and treatment, such as financial constraints and accessibility, in order to maximize the impact of public health interventions. Additionally, the study emphasizes the significance of continuous evaluation and program improvement to ensure long-term effectiveness and sustainability. The Lafiyan Yara project serves as a valuable model for future community-based HIV prevention programs and offers essential lessons on the power of collaboration, community engagement, and evidence-based decision-making in addressing public health challenges.

## Data Availability

The datasets used and/or analysed during the current study are available from the corresponding author on reasonable request.

## Abbreviations

TBA: Traditional birth attendants
VHW: Village Health Workers
PPMV: Patent and Proprietary Medicine Vendors
